# Cigarette smoking dose-response and suicidal ideation among young people in Nepal: a cross-sectional study

**DOI:** 10.3126/nje.v10i1.28277

**Published:** 2020-03-30

**Authors:** Brijesh Sathian, Ritesh G. Menezes, Mohammad Asim, Ahammed Mekkodathil, Jayadevan Sreedharan, Indrajit Banerjee, Edwin R. van Teijlingen, Bedanta Roy, Supram Hosuru Subramanya, Magdy A. Kharoshah, Elayedath Rajesh, Ullasa Shetty, M. Arun, Pradhum Ram, Vinod K Srivastava

**Affiliations:** 1 Surgery Department, Trauma Surgery, Hamad General Hospital, Doha, Qatar; 2 Forensic Medicine Division, Department of Pathology, College of Medicine, Imam Abdulrahman Bin Faisal University, Dammam, Saudi Arabia; 3 College of Medicine, Gulf Medical University, Ajman, United Arab Emirates; 4 SSR Medical College, Belle Rive, Mauritius; 5 Centre for Midwifery, Maternal and Perinatal Health, Bournemouth University, Bournemouth, UK; 6 Department of Physiology, Quest International University Perak (QIUP), city Campus, Ipoh, Perak Darul Ridzuan, Malaysia; 7 Department of Medical Microbiology, Manipal College of Medical Sciences, Pokhara, Nepal; 8 Forensic Medicine Centre, Dammam, Saudi Arabia; 9 School of Behavioural Sciences, Mahatma Gandhi University, India; 10 Department of Forensic Medicine, A. J. Institute of Medical Sciences, Mangalore, India; 11 Department of Forensic Medicine, JSS Medical College, JSS Academy of Higher Education and Research, Mysore, India; 12 Division of Cardiovascular Medicine, Emory University Hospital, Atlanta, GA, USA; 13 Prasad Institute of Medical Sciences, Lucknow, India

**Keywords:** Suicidal ideation, Tobacco, Smoking, Mental health, Youth, South Asia

## Abstract

**Background:**

Worldwide, tobacco smoking is a major risk factor for morbidity and early mortality among adult population. The present study aimed to find out the association between current smoking and suicidal ideation among young people in Nepal.

**Materials and Methods:**

A cross-sectional questionnaire-based survey was carried out among 452 youths from Pokhara, Nepal. The present study included both genders (age 18-24 years) who were smokers as well as non-smokers.

**Results:**

Across the study period, 452 participants were identified after matching for age, and sex (226 in the smoking group and 226 in the non-smoking group). The mean age of participants was 21.6±1.2 years and 58.8% were males. The overall rate of suicidal ideation in our cohort was 8.9%. Smokers were slightly more likely to report suicidal ideation than non-smokers (aOR 1.12). The risk of developing suicidal ideation was 3.56 (95% CI 1.26-10.09) times more in individuals who smoked greater than 3.5 cigarettes per week (p=0.01).

**Conclusion:**

The rate of suicidal ideation was slightly higher among smokers and a dose-response relationship was identified with the number of cigarettes smoked per week. Being aware of the link between smoking and suicidal ideation may help health care professionals working with young people to address more effectively the issues of mental well-being and thoughts about suicide.

## Introduction

Worldwide, tobacco intake is a leading risk factor for early mortality, with a projected figure of 6.4 million deaths in 2015 [1]. Smoking is identified as a potential risk factor for mortality due to lung cancer, respiratory disorders, and cardiovascular diseases [1]. Non-communicable diseases (NCDs) account for about 43% of the total deaths in Nepal [2]. This substantial burden of mortality may be linked to the higher smoking rates among young people [1]. In 2015, the age-standardised prevalence of smoking was found to be 12.7% in females and 27.4% in males in Nepal [1]. The prevalence of smoking was 12% among the age group of 20-29 years and 4.3% among the 14-19 age group, despite the fact that the sale of cigarettes to minors (under 18 years of age) is a legal offense in Nepal [3].

Suicide contributes to 1.4% of the global burden of disease, and the majority of cases belong to the age group of 15-35 years [4]. Several studies have demonstrated that nicotine or tobacco dependence is associated with suicide [5-10]. In addition, some epidemiological studies also reported a higher incidence of suicidal ideation among current but not former smokers [8-10]. Nevertheless, the mechanism by which smoking increases the suicidal behaviour remains unclear [8-10]. A meta-analysis by Poorolajal and Darvishi reported that current smokers had 2.05 times increased risk of suicidal ideation when compared to non-smokers [11]. To date, there are no studies in Nepal on suicidal ideation in current cigarette smokers. Therefore, the present community-based study aimed to assess the association of current cigarette smoking and suicidal ideation among young people in the Pokhara Valley, western Nepal.

## Methodology

### Study design and participants

A cross-sectional questionnaire-based survey was carried out among 452 youths from Pokhara, the second-largest city in Nepal from 1^st^ January 2012 to 31^st^ April 2012. Pokhara has 32 wards and a population of 446,764. The survey was conducted in the selected households of wards (Armala, Bhalam, Hemja, and Pokhara sub-metropolitan city) [12], where the Department of Community Medicine of Manipal College of Medical Sciences, Pokhara, Nepal conducts routine field visit programmes for undergraduate medical students. The questionnaire was administered personally by one of the investigators visiting households in the study area. The study group comprised non-smokers and smokers from both the sexes aged 18 to 24 years and who completed the questionnaire in the presence of the investigator.

Non-probability sampling was used, and data were collected using a self-administered structured questionnaire with closed or multiple-choice questions, which offered the respondent a set of predetermined answers [13]. It was constructed based on the standardized WHO (World Health Organisation) Global Youth Tobacco Survey (GYTS) questionnaire [14]. The GYTS questionnaire consists of questions on the prevalence of cigarette smoking and other tobacco use among young people, knowledge, and attitudes of young people towards cigarette smoking, access to cigarettes, the role of the media and advertising in young people’s use of cigarettes, environmental tobacco smoke, tobacco-related school curriculum, and cessation of cigarette smoking. The present study questionnaire was constructed using the questions related to the knowledge of youths towards cigarette smoking, the prevalence of cigarette smoking, and the number of cigarettes smoked per week. However, the other questions of the GYTS questionnaire were not considered in the present study. The questions were intending to collect information about parental smoking status, smoking status of friends, and watching advertisements related to smoking and awareness about passive smoking were amended [14]. The exclusion and amendment were done according to the suggestions by a panel of public health experts in Nepal. The modified questionnaire was translated into Nepali by two individuals who were experts in both English and Nepali. Then the questionnaire was back-translated into English by another two language experts and validated.

Recent suicidal ideation (over the past 12 months) was assessed with the help of four questions in the General Health Questionnaire (GHQ28) pertaining to suicide [15,16]. These questions were validated and found to have similar sensitivity in detecting suicidal ideation as other suicidal intent scales [17]. The four questions were: ‘‘Have you recently found yourself wishing that you were dead and away from it all?’’; ‘‘Have you recently felt that life is not worth living?’’; ‘‘Have you recently had thoughts of the possibility that you might do away with yourself?’’; and ‘‘Have you recently found the idea of taking your own life coming into your mind?’’ Responses to the questions were scored on a Likert-type scale with the ﬁrst two questions having responses of, ‘‘Not at all/No more than usual/Rather more than usual/Much more than usual’’ and the latter two having responses of, ‘‘Deﬁnitely not/I don’t think so/Has crossed my mind/Deﬁnitely has’’. If the response was ‘‘Rather more than usual/Much more than usual’’ and/or ‘‘Has crossed my mind/Deﬁnitely has’’ to any of the four questions, then the responses were considered as positive, and the subject was considered to have suicidal ideation.

### Inclusion criteria

The present study included non-smokers and smokers from both the sexes aged 18 to 24 years and who have completed the questionnaire in the presence of the investigator.

### Exclusion criteria

Those who did not complete the questionnaire.

### Outcome variable

The outcome variable was suicidal ideation.

### Explanatory variable

Factors that were taken into account at the individual level were age, sex, smoking status, parental smoking status, smoking status of friends, knowledge about passive smoking, and watching advertisements related to smoking.

Each respondent was classified as “tobacco smoker”, if the response to “Do you currently smoke cigarettes?” was “yes“.

### Ethics

Ethics committee approval was obtained before the commencement of the study from the Institutional Research and Ethics Committee of Manipal College of Medical Sciences, Pokhara, Nepal (affiliated with Kathmandu University), which is authorized by the Nepal Health Research Council. Information regarding the study was provided to the participants, and the informed verbal consent from each participant was taken in the presence of two witnesses without any competing interests prior to the distribution of the questionnaire, and the completed questionnaire was collected on the same day. It was made very clear to the participants that they had a free choice to decide whether or not to participate. The research was conducted in accordance with the Declaration of Helsinki [18].

### Sample size calculation

The sample size calculation was based on the data from a pilot study with 100 participants. For a 95% confidence interval and, significance level α = 5%, P = 10%, Q = 90%, allowable error = 4, our required sample size in each group was 217, where P is the percentage of suicidal ideation among the smokers. But we had collected information from 961 participants; among them, 30.9% were smokers [19]. In order to eliminate the confounding effect of age and gender on smoking, the present study performed 1:1 matching for the age and sex to get identical cohorts of smokers and non-smokers.

### Data management and statistical analysis

The data were analyzed using Statistical Package for the Social Sciences (SPSS) for Windows Version 21.0 (SPSS Inc; Chicago, IL, USA). Among the non-smokers, matching for age and sex was used to identify the control group for the analysis to overcome the influence of potential confounders for the outcome. The Chi-square test and Student’s t-test were used to observe the relationship between different variables. The strength of the relationship between suicidal ideation and smoking was observed using the binary logistic regression analysis. Box-plot was used to depict the association between suicidal ideation status and the number of cigarettes per week. The receiver operating characteristic curve was used to determine the area under the curve, sensitivity, specificity, and cut-off level of the number of cigarettes per week to predict suicidal ideation. We have calculated the adjusted odds ratios (aOR) and their 95% confidence intervals (95% CI). For all calculations p<0.05 was considered as statistically significant.

## Results

### Overall study characteristics

**Figure 1:**
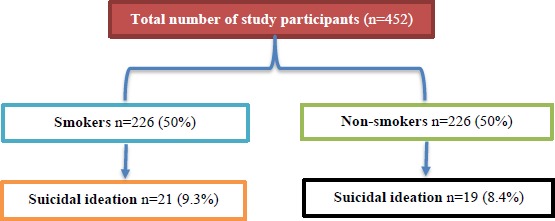
Flow diagram showing study design and outcomes

Across the study period, 452 participants were identified after matching for age and sex (226 in the smoking group and 226 in the non-smoking group). The mean age of participants was 21.6±1.2 years, and 58.8% were males. Overall, the frequency of parental smoking and smoking by friends was found to be 36% and 62.2%, respectively. Also, 95.4% of the participants had seen advertisements related to smoking, and 40.5% were aware of passive smoking. The overall rate of suicidal ideation in our cohort was 8.9%. Figure 1 shows the study design and outcomes. The matching yielded two similar cohorts with comparable age and gender with respect to smoking status (Table 1).

### Comparison between study variables in the non-smoking and smoking groups

Table 1 shows that parental smoking habits significantly affected the smoking habit of their children (p=0.001). Besides this, smoking status of friends also influenced the smoking habit of the participants to some extent (p=0.003). Smokers were less likely to be aware of the risks of passive smoking than non-smokers (p=0.001). Around 95% of the participants in both groups had watched advertisements related to smoking.

### Comparison between study variables based on suicidal ideation status

Table 2 shows that smokers were slightly more likely to report suicidal ideation than non-smokers [aOR 1.12 (0.58-2.18)]. The rate of suicidal ideation was higher in 22-24 years age group and male gender.

### Subgroup wise comparison of smoking and suicidal ideation

In males, there was a higher risk of suicidal ideation among smokers (OR 1.137; 95% CI 0.563-2.298; P=0.72). However, there was no association between suicidal ideation and smoking in females (OR 1.00 95% CI 0.14-7.25; p=1.00).

### Comparison of the number of cigarettes based on suicidal ideation status

**Figure 2:**
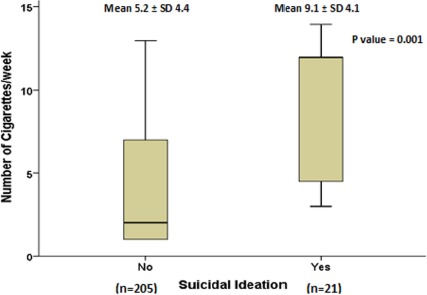
Suicidal ideation by the number of cigarettes per week

**Figure 3:**
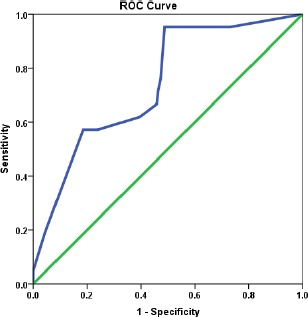
ROC analysis for suicidal ideation and the number of cigarettes per week

Figure 2 depicts that the average number of cigarettes smoked per week was significantly higher among those who had suicidal ideation (p=0.001). The ROC curve showed prediction of suicidal ideation based on the number of cigarettes per week (AUC: 0.741; 95% CI: 0.63-0.85, p=0.001; cut off value: 3.5; sensitivity: 76.2%; specificity: 52.7%) (Figure 3). The risk of developing suicidal ideation was 3.56 (95% CI 1.26-10.09) times more in individuals who had smoked greater than 3.5 cigarettes per week (p=0.01).

## Discussion

### Smoking versus other factors

The age standardised prevalence of smoking was 27.4% and 12.7% in males and females, respectively [1]. Sreeramareddy et al. [20] reported a similar pattern among medical students in Pokhara, Nepal. Aryal et al. found a high prevalence of smoking (72.4%) among college students of Kathmandu Valley, Nepal; males were more likely to smoke than females [21]. Studies from China and Nepal showed the prevalence rates of smoking among students to be age-related [21, 22]. Consistent with our findings, Engels et al. reported that the smoking status of parents and friends influenced the smoking behaviour of the participants [23]. Parents’ low education status and smoking habit had a direct impact on developing the smoking habit among their off-springs [24, 25].

### Suicidal ideation versus age and sex

In our study suicidal ideation was 7.5% in the 18-21 years age group, increasing to 10.4% in the 22-24 group, whilst there was a marked difference between men (13.5%) and women (2.2%). Evans et al. reported the following prevalence rates of suicidal ideation in a meta-analysis of over a half million adolescents: recent suicide ideation (21.3%), past month (30.7%), past year (19.3%), and lifetime (29.9%) [26]. These figures are higher than the findings in our study (8.9%). Large American epidemiological studies also reported the lifetime prevalence figures for adults as 11.18 to 16.52% [27, 28]. In addition, Fergusson et al. reported the lifetime prevalence of suicidal ideation in boys aged 16 and 21 as 9.5% and 24.5%, respectively [29].

In this study, suicidal ideation was higher in men than in women. On the contrary, studies conducted in Korea and Uganda found the prevalence of suicidal ideation to be slightly higher among females [30, 31]. However, other studies did not account for the sex dissimilarity for suicidal ideation [32].

### Smoking and suicidal ideation

In this study, 9.3% of the smokers had suicidal ideation when compared to 8.4% non-smokers. Possible justifications of suicide in smokers include: smoking causes depression, changes in brain chemistry, and the occurrence of lung cancer, which independently leads to considering suicide. Poorolajal and Darvishi reported in a meta-analysis that smoking is related to suicidal ideation (OR 2.05), which is slightly higher as compared to our findings [11]. At present, it is unclear as to whether smoking cessation increases the risk of suicide during the initial phase of tobacco withdrawal [8, 10, 33, 34]. Although the mechanism by which smoking may increase the risk of suicidal behaviour is not known, several studies conducted in various populations, ethnic groups, and ages consistently report associations between smoking and suicide [35-40].

### Policy implications

In Nepal, tobacco is used in a variety of ways. The results of our study point towards the need for targeted approaches in tobacco control. To achieve comprehensive control, strategies of preventing tobacco use must be applied not only to today’s youth but also to future generations. This study indicates that any programme to counter the problem of suicidal ideation among youth should not be limited to college activities but also be extended to deal with adverse life events and stress at home and in the family. Research highlighting factors associated with suicidal ideation could feed into policy development [15, 16, 41]. Mass media campaigns are a prominent tool in promoting smoking cessation among adults, and some have been found to be effective in promoting cessation-related outcomes [42-44]. Cessation-focused campaigns have employed a relatively wide variety of message themes, including: ‘why to quit’, ‘how to quit’, and ‘limiting the tobacco industry’.

## Limitation of the study:

The cross-sectional design of the present study limits the ability to draw causal inferences. However, we have done 1:1 matching for age and sex to improve the quality of inference in our cohort. We could not establish other possible associations since we have not collected the information on other potential co-variants such as socioeconomic status, psychological, or psychiatric factors. Further, longitudinal studies are required to assess the relationship between smoking and suicidal ideation. Although the findings were not established on a clinical sample, this study may have clinical implications. A clinical trial is needed to rule out smoking as a risk factor for suicidal behaviour.

The number of cigarettes smoked per week could be underestimated due to recall bias, and people sharing and gifting cigarettes which also make estimating exact numbers smoked very difficult [45].

## Conclusion

The rate of suicidal ideation was slightly more among smokers, and a dose-response relationship was identified with the number of cigarettes smoked per week. Being aware of the link between smoking and suicidal ideation may help health care workers, teachers, and other relevant professionals working with young people to address more effectively the issues of mental well-being and thoughts about suicide.

### Future scope of the study:

As the present study is cross-sectional in nature, further randomised controlled trials are needed to validate the dose-response relationship between the number of cigarettes/day and suicidal ideation.

### What is already known on this topic?

The existing evidence suggests that the current smokers were at higher risk of suicidal ideation in comparison to non-smokers.

### What this study adds:

This study demonstrated a dose-response relationship between the number of cigarettes/week and suicidal ideation.

## Figures and Tables

**Table 1:** Cross-tabulation of smoking and other factors

Variable	Non-smokers(n=226)	Smokers(n=226)	p value
**Age (mean ± SD); years**	21.6±1.3	21.6±1.3	1.00
**Gender**			
**Males**	133 (58.8%)	133 (58.8%)	1.00
**Females**	93 (41.2%)	93 (41.2%)	
**Parental smoking**	36 (15.9%)	127 (56.2%)	0.001†
**Smoking by friends**	125 (55.3%)	156 (69.0%)	0.003†
**Have you seen advertisements related to smoking?**	215 (95.1%)	216 (95.6%)	0.8
**Are you aware of passive smoking?**	111 (49.1%)	72 (31.9%)	0.001†

† Statistically significant (p<0.05)

**Table 2:** Cross-tabulation of suicidal ideation and other factors

Variable	No-suicidal ideation (n=412)	Suicidal ideation (n=40)	p value	aOR (95% CI)
**Age**				
**18-21 years**	222 (92.5%)	18 (7.5%)	0.28	1.39 (0.7-2.7)
**22-24 years**	190 (89.6%)	22 (10.4%)		
**Gender**				
**Females**	182 (97.8%)	4 (2.2%)	0.001 †	1
**Males**	230 (86.5%)	36 (13.5%)		7.1 (2.5-20.3)
**Smoking**				
**No**	207 (91.6%)	19 (8.4%)	0.74	1
**Yes**	205 (90.7%)	21 (9.3%)		1.12 (0.58-2.18)

† Statistically significant (p<0.05)
